# Expression profiling of long noncoding RNAs in neonatal and adult mouse testis

**DOI:** 10.1016/j.dib.2015.06.004

**Published:** 2015-06-23

**Authors:** Jin Sun, Ji Wu

**Affiliations:** a Key Laboratory for the Genetics of Developmental & Neuropsychiatric Disorders (Ministry of Education), Bio-X Institutes, Shanghai Jiao Tong University, Shanghai, China; b Key Laboratory of Fertility Preservation and Maintenance of Ministry of Education, Ningxia Medical University, Yinchuan, Ningxia, China; c National-Local Joint Engineering Research Center of Biodiagnostics and Biotherapy, Xi’an Jiaotong University, Xi’an, China

**Keywords:** Testis development, Spermatogenesis, LncRNA, Microarray

## Abstract

In recent years, advancements in genome-wide analyses of the mammalian transcriptome have revealed that long noncoding RNAs (lncRNAs) is pervasively transcribed in the genome and an increasing number of studies have demonstrated lncRNAs as a new class of regulatory molecules are involved in mammalian development (Carninci et al. (2005); Fatica and Bozzoni (2014)), but very few studies have been conducted on the potential roles of lncRNAs in mammalian testis development. To get insights into the expression patterns of lncRNA during mouse testis development, we investigated the lncRNAs expression profiles of neonatal and adult mouse testes using microarray platform and related results have been published (Sun et al., PLoS One 8 (2013) e75750.). Here, we describe in detail the experimental system, methods and validation for the generation of the microarray data associated with our recent publication (Sun et al., PLoS One 8 (2013) e75750.). Data have been deposited to the Gene Expression Omnibus (GEO) database repository with the dataset identifier GSE43442.

## 1 Specifications

**Table:** 

Organism/cell line/tissue	Mus musculus
Sex	Male
Sequencer or array type	Arraystar Mouse Stringent LncRNA microarray
Data format	Raw data: TXT files, normalized data: SOFT, MINIML, and TXT TXT files
Experimental factors	Neonatal (6-day-old) and adult (8-week-old) C57/BL6 mouse testis
Experimental features	Microarray comparison was preformed to identify genes differentially expressed in neonatal (6-day-old) and adult (8-week-old) mouse testis
Consent	N/A
Sample source location	C57/BL6 mouse, Shanghai, China

## 2 Direct link to deposited data

Microarray data is accessible under the following link: http://www.ncbi.nlm.nih.gov/geo/query/acc.cgi?acc=GSE43442

## 3 Experimental design, materials and methods

### 3.1 Collection of testis samples

Twenty-one neonatal (6-day-old) and six adult (8-week-old) male C57BL/6 mice were purchased from SLAC Laboratory Animal Co., Shanghai, China. Mice in each age group were divided into three groups to provide three biological replicates for microarray analysis. Mice were sacrificed by cervical dislocation. Whole testes were surgically removed from mice and were immediately snap-frozen in liquid nitrogen and ground to a fine powder with mortar and pestle that was pre-cooled with liquid nitrogen and then Trizol reagent (Invitrogen) was added to continue grinding.

### 3.2 RNA extraction

Total RNA was extracted according to manufacturers instructions of Trizol reagent. RNA quantity and quality were measured by NanoDrop ND-1000 spectrophotometer (Thermo Scientific). RNA integrity and genomic DNA contamination were assessed by denaturing agarose gel electrophoresis and Bioanalyzer 2100 (Agilent Technologies).

### 3.3 RNA labeling and array hybridization

Sample labeling and array hybridization were performed according to the Agilent One-Color microarray-based gene expression analysis protocol (Agilent Technology). Briefly, for RNA labeling, 1 μg of total from each sample was labeled with Cy3-dCTP using RNA Spike-In Kit (Agilent, 5188-5282) and Quick Amp labeling kit (Agilent, 5190-0442), the labeled cRNAs were purified by RNAeasy Mini Kit (Qiagen), the concentration and specific activity of the labeled cRNAs (pmol Cy3/μg cRNA) were measured by NanoDrop ND-1000 Spectrophotometer (Thermo Scientific). For array hybridization, 1 μg of each labeled cRNA was fragmented by adding 11 μl 10×Blocking Agent and 2.2 μl of 25×Fragmentation Buffer (Agilent), then heated the mixture at 60 °C for 30 min, finally 55 μl 2×GE Hybridization buffer (Agilent) was added to dilute the labeled cRNA. 100 μl of hybridization solution was dispensed into the gasket slide and assembled to the lncRNA expression microarray slide (Mouse Stringent LncRNA microarray, 4×44 K, ArrayStar). The slides were incubated for 17 h at 65 °C in Agilent’s SureHyb Hybridization Chambers. After hybridization, microarrays were washed 1 min at room temperature with GE Wash Buffer 1 (Agilent) and 1 min with Wash buffer 2 (Agilent) at 37 °C, then dried immediately by brief centrifugation. The hybridized arrays were washed, fixed and scanned with using the Agilent DNA Microarray Scanner (part number G2505B).

### 3.4 Data analysis

**Fig. 1:**
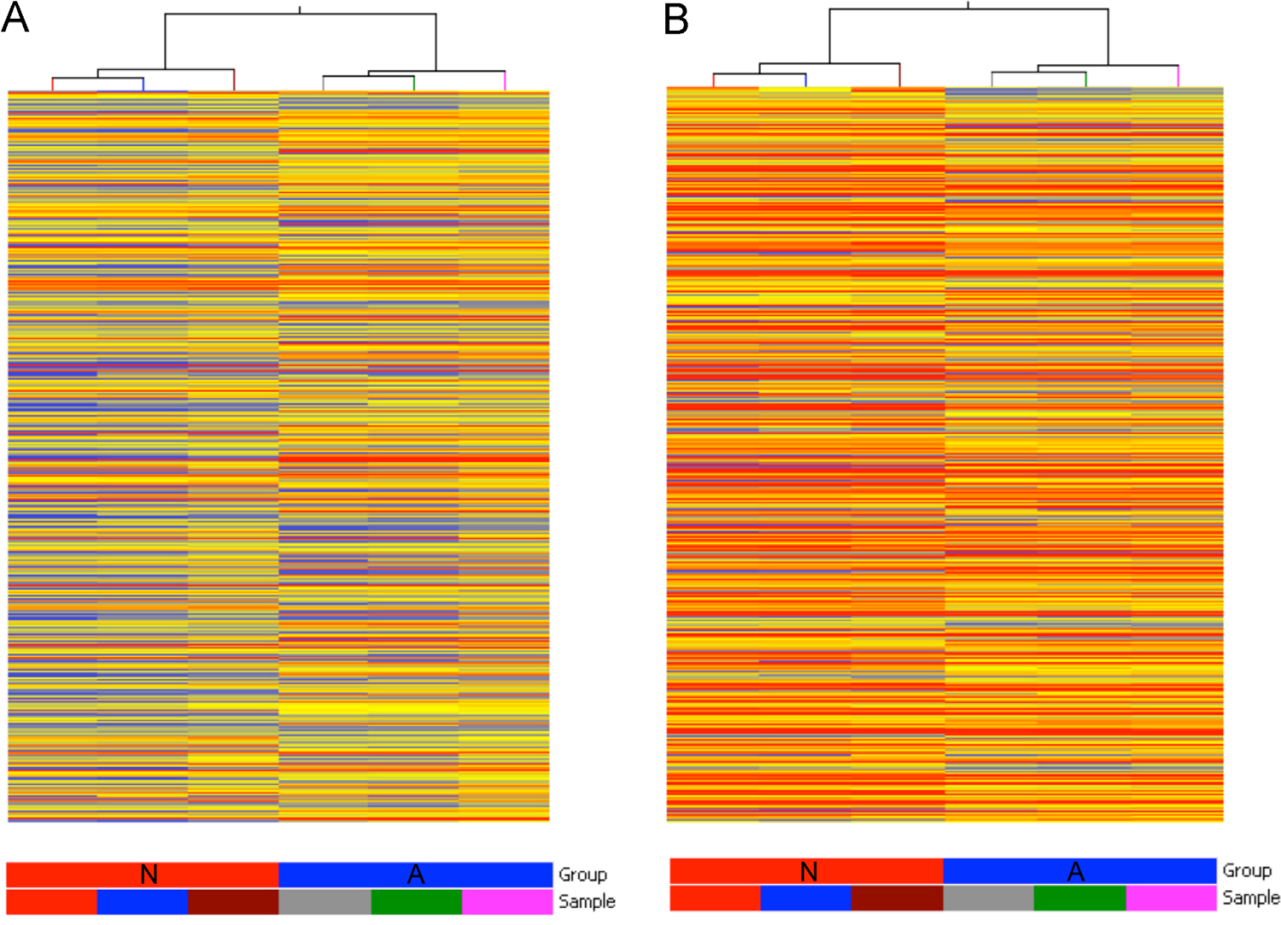
Hierarchical Clustering for All Targets Value in Group N (neonatal) and Group A (Adult). (A) indicates lncRNAs and (B) indicates mRNAs. “Red” indicates high relative expression, and “blue” indicates low relative expression.

Agilent Feature Extraction software (version 10.5.1.1) was used to extract raw data from scanned array images. Median normalization and subsequent data processing were performed with using the GeneSpring GX v11.0 software package (Agilent Technologies). After median normalization of the raw data and low intensity filtering, lncRNAs and mRNAs that at least 1 out of 6 samples have flags in Present (“All Targets Value”) were chosen for further data analysis. The quality of lncRNA and mRNA was assessed by Box-Plot and Scatter-Plot. The Box Plot is commonly used for comparing the distributions of the intensities from all samples. After normalization, the distributions of log2-ratios among all tested samples are nearly the same. The Scatter-Plot is a visualization method used for assessing expression variation (or reproducibility) between the two compared arrays. Differentially expressed lncRNAs and mRNAs were screened through performing a Volcano plot filtering between the two samples. The threshold is Fold Change >=5.0 and *p*-value<=0.05 (Student’s *t*-test). Hierarchical Clustering was performed using the Agilent GeneSpring GX software (version 11.0) (Fig. 1). GO (Gene Ontology) analysis and Pathway analysis was performed using Database for Annotation, Visualization and Integrated Discovery (DAVID) [4]. Raw and processed microarray data have been deposited to the Gene Expression Omnibus (GEO) database in the National Center for Biotechnology Information (NCBI), and can be accessed by the GEO accession number, GSE43442.

### 3.5 Validation of microarray data

**Fig. 2:**
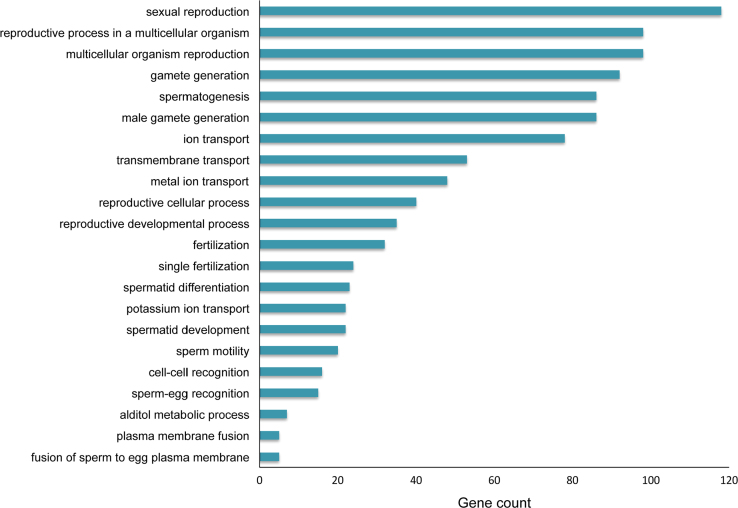
Significantly enriched Gene ontology (GO) terms (*p*<0.05) in the up-regulated genes in adult testis.

To validate the microarray data, we investigated the expression level of eight differentially expressed lncRNAs in neonatal (6-day-old) and adult (8-week-old) mouse testis using quantitative real-time PCR (qRT-PCR). The results clearly showed that there was a excellent correlation (Spearman coefficient rho=0.952, *p*<0.01, *n*=8) between the microarray data and the qRT-PCR data (Table 1), indicating that the microarray results were reliable. The primers used for qRT-PCR see Table 2. In addition, GO analysis revealed that up-regulated mRNAs in adult testis was significantly enriched in reproduction-related GO terms, such as sexual reproduction, multicellular organism reproduction, gamete generation and spermatogenesis and so on (shown in Fig. 2), indirectly indicating the microarray results were reliable.

## 4 Discussion

Genome-wide analyses of the mammalian transcriptome revealed that lncRNAs is pervasively transcribed in the genome and accumulating studies have demonstrated that lncRNAs as novel regulatory molecule plays critical roles in mammalian development [1,2], but little is known about the potential roles of lncRNAs in mammalian testis development. Here, we described a dataset composed of lncRNA and mRNA expression profiling of neonatal (6-day-old) and adult (8-week-old) mouse testis. With this experiment, we were able to show 3025 of lncRNAs and 5964 of mRNA are differentially expressed between neonatal and adult mouse testes. Some known haploid male germ cell-specific lncRNAs was also found differentially expressed in this study, for example, *Aldoart2*, *Speer5-ps1* and *Speer9-ps1* [5,6]. The dynamic change of lncRNA expression during mouse testis post-natal development indicated that lncRNAs might play crucial roles in mammalian testis development and spermatogenesis. Thus, this experiment provides a solid foundation for the identification and characterization of key lncRNAs involved in testis development or spermatogenesis.

## Conflict of interest

The authors have no conflicts of interest.

## Figures and Tables

**Table 1:** Validation of microarray results by qRT-PCR [3].

LncRNA ID	Microarray		qRT-PCR	
	Fold change a	*p* Value b	Fold change a	*p* Value b
AK011865	−14.700	0.004	−4.018	0.010
AK006530	3,718.218	5.11E−04	172,187.456	0.027
AK053216	−9.943	0.008	−5.362	0.023
AK018942	14,097.462	2.86E−04	26,493.007	0.142
AK006015	45.452	2.10E−04	68.228	0.032
AK004447	−40.531	0.002	−3.540	0.038
AK033245	−17.668	0.012	−7.105	0.047
AK140218	6.516	0.002	7.806	0.043

a Values indicate the absolute fold-change between paired samples (adult to 6-day-old ratio) detected by microarray or qRT-PCR; negative value indicates down-regulation and positive value indicates up-regulation.

b *p* Value was calculated by the Student’s *t*-test (paired).

**Table 2:** List of primers used in the validation of microarray results by qRT-PCR.

Gene symbol	Sequence (5′–3′)		Amplicon length (bp)
	Forward	Reverse	
AK011865	CCACTTCAAATGGGGAGGGT	GTGATTTGGGACACTGGAGAGAC	182
AK006530	CAAGTGCCTGGGATTGTGGATAC	CAGCCCACTGTCCAGGTCATCTC	211
AK053216	GCCCAAGCGGTCACTCAGTATCA	AGTGGTTCAAGTTACTGCCGCTG	287
AK018942	GCCTTTCCCAAATGTCTGTTCCT	TCAGGTTCAGGGAGTGCTTCTTT	245
AK006015	GCCACACCACCATCCACTATT	GGAGGCGAAACCAGGTAGATT	161
AK004447	TGTAGCATCAAACATTCACGGA	GACAGAAGGACACAGGGCAAAC	196
AK033245	CAAACCCGCAGTTCTTCTCCTT	AGTGCCAGCCCGAGTTGTTC	205
AK140218	TTTACAAAAGGGAAAACTGAGGC	TAGGCTGATAAAGGGCTCAAAGTT	196
GAPDH	GTCGTGGAGTCTACTGGTGTC	GAGCCCTTCCACAATGCCAAA	240

## References

[1] Carninci P., Kasukawa T., Katayama S., Gough J., Frith M.C. (2005). The transcriptional landscape of the mammalian genome. Science.

[2] Fatica A., Bozzoni I. (2014). Long non-coding RNAs: new players in cell differentiation and development. Nat. Rev. Genet..

[3] Sun J., Lin Y., Wu J. (2013). Long non-coding RNA expression profiling of mouse testis during postnatal development. PLoS One.

[4] Huang da W., Sherman B.T., Lempicki R.A. (2009). Systematic and integrative analysis of large gene lists using DAVID bioinformatics resources. Nat. Protoc..

[5] Vemuganti S.A., Bell T.A., Scarlett C.O., Parker C.E., de Villena F.P. (2007). Three male germline-specific aldolase A isozymes are generated by alternative splicing and retrotransposition. Dev. Biol..

[6] Spiess A.N., Walther N., Muller N., Balvers M., Hansis C. (2003). SPEER—a new family of testis-specific genes from the mouse. Biol. Reprod..

